# Effect of Peat Intervention on Pain and Gait in Patients with Knee Osteoarthritis: A Prospective, Double-Blind, Randomized, Controlled Study

**DOI:** 10.1155/2020/8093526

**Published:** 2020-01-20

**Authors:** Myeongkyu Kim, Kyu Hoon Lee, Seung Hoon Han, Sung Jae Lee, Choong-Gon Kim, Jae Ho Choi, Sun Hee Hwang, Si-Bog Park

**Affiliations:** ^1^Department of Rehabilitation Medicine, Hanyang University College of Medicine, 222, Wangsimni-ro, Seongdong-gu, Seoul, Republic of Korea; ^2^Department of Rehabilitation Medicine, Hanyang University Guri Hospital, 153, Gyeongchun-ro, Guri-si, Gyeonggi-do, Republic of Korea; ^3^Department of Integrative Medicine, Korea University School of Medicine, 73, Goryeodae-ro, Seongbuk-gu, Seoul, Republic of Korea; ^4^Marine Ecosystem Research Center, Korea Institute of Ocean Science & Technology, 385, Haeyang-ro, Yeongdo-gu, Busan, Republic of Korea; ^5^Ocean Science and Technology School, Korea Maritime and Ocean University, Rbiotech Co., 727, Taejong-ro, Yeongdo-gu, Busan, Republic of Korea; ^6^Medical Business Department, Corporate Research Institute, Rbiotech Co., Ltd., 2-409, 775, Gyeongin-ro, Yeongdeungpo-gu, Seoul, Republic of Korea

## Abstract

**Methods:**

Knee osteoarthritis patients with Visual Analog Scale (VAS) of 3 or more and Kellgren–Lawrence osteoarthritis grades 1 to 3 were included. Patients with history of intraarticular injection treatment were excluded. Forty-one participants were randomly allocated to the peat intervention group (*n* = 22) or the hot-pack-only control group (*n* = 19). Peat and hot pack were applied to both knees of each group of patients. Each intervention session lasted 20 minutes, and eight sessions were completed over five days. VAS, serum cartilage oligomeric matrix protein (COMP), and gait parameters were evaluated before and after the whole interventions.

**Results:**

VAS in the peat group decreased from 6.000 to 3.409 after intervention (*p* < 0.001) and also decreased in the control group from 5.737 to 4.421 (*p* < 0.001). VAS score reduction between two periods was greater in the peat group than that in the control group (*p* < 0.001). There was no significant difference in the serum COMP level in either intergroup or intragroup analysis. In gait analysis, the gait velocity of the peat group increased from 0.781 m/s to 0.873 m/s after intervention (*p*=0.002), while it decreased in the control group. The knee varus/valgus range of motion during gaits was reduced from 11.455° to 8.439° after intervention in the peat group (*p*=0.006).

**Conclusions:**

This study showed that peat can be considered as a therapeutic option for pain relief of knee osteoarthritis patients. The reduction in knee joint varus/valgus range of motion and the increase in gait velocity after peat intervention were also identified through this research, which is the first to analyze the effects of peat on gait.

## 1. Introduction

Osteoarthritis (OA) of the knee is a common joint disease in which cartilage is destroyed by recurrent physical damage caused by excessive use or trauma. Progression of the disease is now understood to result from abnormal remodeling of affected joint tissues and to involve various inflammatory mediators [1]. As the average human life expectancy increases, the number of patients with this disease is increasing. Disease-related chronic pain and impaired function restrict physical activities and increase medical expenses in those affected by knee OA, which are a huge burden for both patients and society [2, 3].

The primary symptoms of knee OA are joint pain and stiffness. As articular pathology progresses, the patient complains of reduced range of motion, tenderness, and joint swelling. Advanced stages involve joint deformity, weakness of quadriceps muscles, and impaired proprioceptive acuity within the knee [4]. Older adults with knee OA often experience severely decreased quality of life compared with healthy controls [5]. The pathogenesis of OA is multifactorial and includes articular cartilage degeneration, synovial inflammation, thickening of subchondral bone, and ligament and meniscus damage [6, 7]. Many inflammatory mediators, proteases, and fibroblast growth factor signaling pathways are also involved in progression of knee OA [8]. Knee joint misalignment and biomechanical modification also affect the evolution of OA. Kinematic gait analyses of OA patients have shown that the knee joint varus angle during the stance phase of gait is greater than in healthy control subjects [9, 10].

In the early stages of disease, nonpharmacologic interventions are the main treatment choices. Intensive weight loss secondary to increased exercise and diet change lessen pain and knee compressive force [11, 12]. The effects of exercise on pain reduction and knee function are similar to those produced by oral nonsteroidal anti-inflammatory drugs (NSAIDs) [13, 14]. When symptoms are not adequately controlled, pharmacologic treatment should be considered. Oral NSAIDs have similar efficacy to topical NSAIDs with respect to pain relief, but clinicians should consider potential gastrointestinal, renal, and cardiovascular complications [15]. Cyclooxygenase-2-selective NSAIDs can be prescribed to patients at high risk of gastrointestinal side effects. Intraarticular corticosteroid injection, oral acetaminophen or duloxetine, balneotherapy, and/or topical capsaicin are also effective therapeutic options [16]. When conservative treatments fail, total joint replacement is recommended but has risks of potential surgical complications and revision operation [17].

Balneotherapy is the use of natural mineral water, gas, and peloid (mud or clay) for therapy, prevention, and rehabilitation [18]. Balneotherapy is a traditional treatment for musculoskeletal disorders in various countries, and many studies have reported its effects on pain in OA patients. Mud-pack therapy, mud-bath therapy, bath therapy, and spa therapy have proven to be effective in reducing pain and NSAID consumption and improving the quality of life in patients with knee OA [19].

Peat is one of the main peloids used in balneotherapy and is formed when plant material cannot fully decay due to acidic and anaerobic conditions [20]. Chemical analyses have shown that water-soluble extracts from peat contain various humic substances, such as fulvic and ulmic acids [21]. Application of humic substances to patients with psoriasis, rheumatoid arthritis, wheal and flare reaction, allergic rhinitis, or knee OA has shown positive therapeutic effects, indicating that peat may have anti-inflammatory properties [22]. This is the theoretical basis upon which peat has been used as a therapeutic option for musculoskeletal diseases such as OA and rheumatologic diseases in European countries. However, data regarding the effects of peat on biomarkers of knee OA and biomechanical alterations accompanying disease progression are lacking.

The first objective of this study was to evaluate whether Korean peat has similar pain-relieving effects to those shown in the previous studies. The second objective was to assess the effects of peat intervention on serum COMP. Although some biomarkers have been analyzed in previous studies, this study is the first to investigate the relationship between serum COMP and peat intervention. The third objective of this study was to evaluate the biomechanical effects of peat intervention on the gait of knee OA patients. This is the first study in which gait analysis was utilized to assess the effects of peat intervention on ambulatory parameters.

## 2. Materials and Methods

### 2.1. Participants

Patients who met the following criteria were included in the study: knee pain for more than three months; diagnosis of knee OA according to the American College of Rheumatology (ACR) classification criteria [23]; a VAS score for pain of 3 or more; a Kellgren-Lawrence osteoarthritis grade [24] 1 to 3.

Patients meeting the inclusion criteria were excluded from the study if they had any of the following conditions: possibility for injury or exacerbation of previous injury during application of thermal therapy to the knee (e.g., acute skin and soft tissue infectious disease, decreased skin sensations, scar tissue, ischemic disease, edema, or malignancy in the area of the knee); allergic reaction to peat; positive rheumatoid factor; suspicious infectious condition (serum white blood cell count over 10,000/mm^3^); cognitive impairment; diseases or conditions that can affect gait parameters, such as Parkinson's disease; and general contraindications for balneotherapy. Patients with history of intraarticular injection treatment (hyaluronic acid and/or glucocorticoid) were also excluded, as such treatments could affect the results of the study.

Patients were openly recruited using posters displayed in Hanyang University Medical Center and Korea University Medical Center in Seoul, Korea, from May to July 2018. Participants received written information about the study and provided signed informed consent prior to enrollment in the study. This study was approved by Hanyang University Medical Center Institutional Review Board.

### 2.2. Peat

Peat was collected from 187 Cheollipo 1-gil, Sowon-myeon, Taean-gun, Chungcheongnam-do, Korea. About 7.5 × 10^4^ kg tons of peat are buried between 50 to 100 cm below the ground surface in this area. Extracted peat was sifted through a 2 mm soil sieve to remove foreign materials and was then dried. The dried 1.4 kg of peat was mixed with 300 mL of distilled water to maintain the appropriate viscosity for easy shaping. The peat was molded in a 24 cm × 30 cm rectangular low-density polyethylene bag to a 1.5 cm thickness and placed into a 27 cm × 40 cm flat pouch made of hemp. The hemp bag had holes large enough to allow water and peat contents to pass through.

The water content of the peat was 68.75%. The heavy metal content was essentially negligible (lead < 0.04 mg/L; arsenic < 0.05 mg/L; cadmium < 0.002 mg/L). The electrolyte concentrations were as follows: potassium = 0.410 ± 0.019 mg/L; sodium = 0.059 ± 0.002 mg/L; calcium = 0.382 ± 0.009 mg/L; and magnesium = 0.203 ± 0.007 mg/L [25].

### 2.3. Protocol

Participants were randomly allocated to the peat intervention group or the hot-pack-only control group. On the screening test day, VAS scoring, body mass index (BMI) test, routine blood tests (including COMP and C-reactive protein (CRP)), knee X-ray, biomechanical gait analysis, and testing for allergy to peat were conducted. Patients were able to complete the screening tests and participate in the study according to the inclusion and exclusion criteria described above. Two weeks after the screening test, those who met the inclusion criteria participated in a five-day course of peat intervention at Taean-gun, Chungcheongnam-do, Korea.

The experimental group received the peat intervention. The peat was heated to 70°C in a low-density polyethylene bag using an infrared warmer; after heating, one side of the bag was removed to allow transfer of peat chemicals to the patient knee. The heated peat was then placed into a hemp pouch, cooled to 40°C, and placed on the patient's knee. An electric thermal pad was applied to maintain the temperature at around 40°C. Skin temperature was measured immediately before and after each intervention session. The same intervention was performed for the control group, but the impermeable low-density polyethylene bag was not removed, preventing the transfer of chemical substances from the peat to the knee. Patients were not aware of which group they were in because the hemp bag covered the polyethylene bag during the intervention.

Two physical therapists performed the intervention, and at least one doctor was present to monitor any unexpected side effects during the program. Each intervention session lasted 20 minutes, and eight sessions were completed over five days. VAS scoring, routine blood testing (with COMP and CRP), and biomechanical gait analyses were conducted before and after the intervention period. Participants were permitted to continue use of oral painkillers during the study. When knee pain worsened during the study, patients were permitted to take additional doses of acetaminophen after notifying the researchers.

### 2.4. Outcomes

Knee pain during the study was evaluated using a VAS. Participants were asked to rate the degree of pain they felt using numbers from 0 (no pain) to 10 (most intense pain) [26]. Serum COMP is a useful biomarker for diagnosis of knee OA, as it is produced by articular chondrocytes in response to cartilage degradation. Serum COMP concentration reflects the degree of cartilage damage, is positively correlated with VAS, BMI, age, and IL-1*β*, and is negatively correlated with disease duration [27, 28].

Biomechanical gait analyses were carried out using a Human Track Gait Analysis System (Rbiotech Co., Ltd., Seoul, Republic of Korea) equipped with a wireless inertial measurement unit (IMU) sensor and stereo camera to capture kinematic and kinetic data. The IMU sensors were attached to each patient's abdomen around the belly, both thighs (10 cm above the upper patellar border), shank (10 cm below the lower patellar border), and dorsum of the foot. Precise calibration was achieved when the patient stood 1 m in front of the device. The patients were asked to walk naturally for 6 m at a self-selected speed while being monitored by video. Gait velocity, cadence, and each lower extremity joint's range of motion during walk in three planes were analyzed automatically.

VAS scoring, serum COMP measurement, and gait analyses (including range of motion of knee joint while walking) were conducted on the screening test day and on the first and last days of the intervention.

### 2.5. Statistical Analysis

Statistical analyses were performed using SPSS (SPSS Inc., Chicago, IL, USA). The Mann–Whitney test was used for intergroup analysis of VAS, COMP, and biomechanical gait results for the peat intervention and control groups. The Wilcoxon signed-rank test was used for intragroup analyses to assess whether there was meaningful change in the parameters between the pre- and postintervention periods. Significance was noted at *p* < 0.05.

## 3. Results

**Figure 1:**
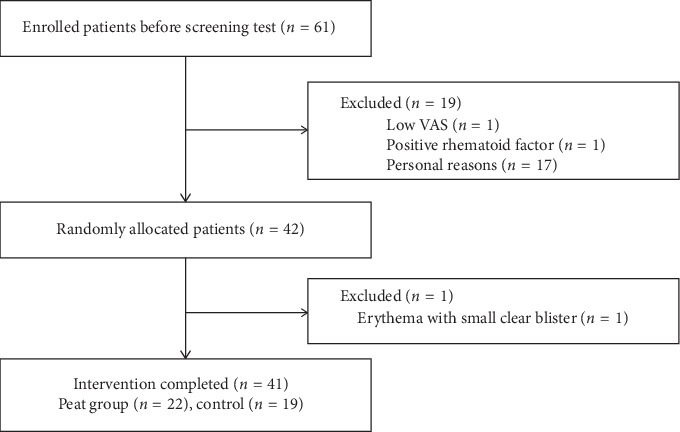
Placement process of the study participants.

Sixty-one patients were enrolled initially. Two patients were excluded from the screening test according to the exclusion criteria, one with a low VAS score and one with a positive rheumatoid factor. Seventeen patients dropped out of the study due to personal reasons. The remaining 42 patients were randomly allocated to the peat intervention or control group. One patient was removed from the study after developing erythema with small clear blisters on the skin of both knees following the first intervention session. The final study population included 22 patients in the peat intervention group and 19 in the control group. The placement process for the study participants is presented in Figure 1.

The demographic characteristics of the study population are presented in Table 1. There was no significant difference in age or BMI between the peat intervention and control groups. Overall, the average age was 65.83 ± 7.61 years; 9 participants were in their 50s, 21 in their 60s, 10 in their 70s, and 1 in 80s. Ten patients were male, and 31 patients were female. Sixteen patients were Kellgren–Lawrence osteoarthritis grade 1, 14 patients were grade 2, and 10 patients were grade 3.

In intragroup analyses, both the peat intervention and control groups showed significant pain relief after the intervention. The VAS score of the peat group decreased from 6.000 to 3.409 and that of the control group decreased from 5.737 to 4.421; each *p* value was less than 0.001. There were no significant differences in the intergroup comparisons in the postintervention period (*p*=0.066); however, VAS score reduction between the two assessment periods was greater in the peat intervention group (2.591 ± 1.333) than in the control group (1.316 ± 0.885), and the difference was significant (*p* < 0.001). There was no significant difference in VAS score between the two groups at the preintervention period. Serum COMP concentration decreased after the intervention in both groups, but the reductions were not statistically significant in either intergroup or intragroup analysis (Table 2).

In gait analysis, the gait velocity of the peat intervention group increased from 0.781 m/s to 0.873 m/s after intervention (*p*=0.002), while that of the control group decreased from 0.772 m/s to 0.727 m/s (*p*=0.180). Intergroup analysis of velocity at the postintervention period showed a significant difference (*p*=0.006). Gait cycle time decreased in both groups, but the reductions were not statistically significant. In the peat intervention group, cadence (i.e., steps per minute) increased significantly from 91.774 steps/min to 95.284 steps/min after the intervention (*p*=0.035). The cadence of the control group increased from 90.327 steps/min to 91.675 steps/min, but the change was not significant (*p*=0.440). The intergroup cadence measurements during the pre- and postintervention periods were not significantly different (Table 3).

Knee varus/valgus range of motion during the assessed gaits was reduced from 11.455° to 8.439° after intervention in the peat group, with a *p* value of 0.006, and from 10.180° to 10.144° in the control group, but these results were not statistically significant (Table 4).

## 4. Discussion

In this study, we assessed the pain-relieving effects of a short-term intervention with peat collected from Taean-gun, Chungcheongnam-do, Korea. As mentioned in the results, we found that the group which received peat intervention had a greater decrease in VAS than the control group with statistical significance. Several studies using VAS as a measure of knee pain have demonstrated the effectiveness of pelotherapy for pain relief [19, 29–32]. In particular, several studies have confirmed that pain relief persists even after long-term follow-up of 6 months or longer [30, 32].

In this study, pain was also reduced in the control group, for which exposure to the peat was prevented by a low-density polyethylene bag. It is possible the pain of the control group was reduced due to the temperature of the applied peat pack (40°C). Heat is postulated to reduce pain through various mechanisms, including gate control, in which heat reduces muscle spasm and increases the pain threshold and blood flow to applied area [33]. Thermal saunas and hydrotherapy elevate serum beta-endorphin concentrations, which play a role in endogenous pain relief [34].

Although the temperature of the peat packs may have reduced the VAS score of both groups, the greater pain reduction in the peat intervention group can be interpreted as a biochemical effect of peat on knee OA. Ortega et al. [29] showed that pelotherapy for 10 days reduced serum IL-1*β*, TNF-*α*, IL-8, IL-6, and TGF-*β* concentrations. Research in a controlled group setting also showed that mud-pack therapy reduced VAS, increased serum IGF-1, and maintained YKL-40 level in patients with knee OA compared to the hot-pack-only control group [30, 31]. Peat contains greater concentrations of humic acids than regular mud; these humic acids are postulated to have immune regulatory effects. It has been shown that application of humic acid extracts from mud to patients with allergic diseases or OA reduced pain and symptoms and improved quality of life [22]. In physiochemical analysis, murine splenic lymphocytes incubated with fulvic acid, one of the main types of humic acid in mud, showed increased uptake of thymidine and enhanced production of reactive oxygen species [35], but other studies have reported mixed effects on inflammatory cytokines [30]. Peat has more abundant lipophilic components than other muds and contains low-molecular weight organic substances composed of fatty acids, as well as hydrophilic substances. Its pharmacological effects are not yet fully understood [36]. Hence, further research to understand the molecular mechanisms of humic acid and the role of lipophilic substances from peat is needed.

COMP is a pentameric noncollagenous glycoprotein and a degradation product of articular cartilage. Its diagnostic value is recognized by the Foundation for the National Institutes of Health (FNIH), along with urinary C-terminal telopeptide and serum hyaluronic acid concentrations [37]. This is the first study to assess the effect of peat intervention on serum COMP concentration.

In this study, we concluded that peat intervention does not have a significant effect on serum COMP concentration during a short-term evaluation period. The peat intervention group experienced only a slight nonsignificant reduction of serum COMP compared to the preintervention period. Serum COMP concentration is a biomarker of early OA progression, as COMP concentration decreases in advanced stages of OA [27], and the duration of disease should therefore be considered when assessing serum COMP. Although serum COMP has good diagnostic and prognostic value, it is not currently included in the ACR classification criteria for knee OA diagnosis. Standard diagnosis and estimation of OA severity require assessment of clinical symptoms, radiological findings, and various biomarkers.

The intervention group in this study showed significantly increased gait velocity after five days of peat intervention compared to their preintervention evaluation. Gait cycle time was reduced in both groups after the intervention, but the reduction was greater in the peat intervention group. Intragroup analysis found cadence to be significantly increased in the peat intervention group but not in the control group. The enhancement in speed appeared to be correlated with decreased gait cycle time and increased cadence, which may have improved due to reduction in pain. In a previous study, two weeks of spa therapy increased gait velocity, cadence, and stride length and decreased pain; however, neither mud nor peat therapy was assessed, and data regarding angular analysis of the lower extremities were not collected [38].

The adduction angle of the knee joint is generally known to be increased in knee OA patients relative to the general population [9]. Previous studies of patients with knee OA have found that the larger is the varus/valgus range of motion of the knee, the lower is the functional ability and that this relationship is stronger in patients with severe varus deformity [39]. In this study, the knee varus/valgus range of motion while walking decreased from 11.455° during the preintervention period to 8.439° during the postintervention period, with a *p* value of 0.006. The range of motion in the control group was not significantly different between the pre- and postintervention periods. This finding indicates that peat intervention reduces the range of motion of the knee joint in the frontal plane and improves dynamic joint stability. However, a previous study suggested that varus/valgus range of motion has little correlation with joint stability parameters such as joint laxity, skeletal alignment, muscle strength, and joint proprioception [40]. For this reason, it is reasonable to assume that multiple factors may have contributed to stabilization of the knee joint. Even though range of motion was significantly reduced in this study, multiple regression analysis is needed to determine which factors affected the reduced range of motion and joint stability.

Several previous studies have shown that knee OA patients reduced gait velocity to reduce the adductor moment [41, 42]. In the peat intervention group, reduction of the varus/valgus range of motion of the knee joint between the pre- and postintervention periods was inversely correlated with the change in gait velocity (*r* = −0.365, *p*=0.016). For this reason, increased gait velocity of the peat intervention group in this study appeared to be related to improved angulation in the frontal plane. This is the first study of the effects of mud therapy on gait parameters and angular distribution of lower extremity joints in knee OA patients.

There are several limitations of this study. First, the peat maturation process was omitted in this research and it was used immediately after the impurity exclusion process. Previous papers noted that matured mud contained more sulfoglycolipids, which are thought to be responsible for the anti-inflammatory properties of thermal mud therapy [43]. In addition, many papers investigating clinical applications of mud in OA patients found relief of pain and inflammatory stress using matured mud [29, 30]. Subsequent studies using mature mud need to be considered. Second, if intervention had been carried out for more than two weeks, better results may have been obtained. The Turkish Rheumatism League has recommended at least two weeks of balneotherapy to maximize its thermal and nonthermal effects [44]. Third, a large amount of peat was required to mold it into the rectangular shape of our peat pack, which some patients found heavy. Different application methods, such as painting, may need to be considered. Fourth, the small sample size and short follow-up period are problems to be addressed in future research.

## 5. Conclusion

This study showed that peat obtained from Taean-gun, Chungcheongnam-do, Korea, can be considered as a therapeutic option for pain relief of knee OA patients. Although short-term peat intervention did not have significant effects on serum COMP, long-term follow-up assessment using several parameters, including pain and OA biomarkers, is needed. The reduction in knee joint varus/valgus range of motion and the increase in gait velocity after peat intervention are meaningful results of our research study, which is the first to analyze the effects of peat intervention on gait parameters.

## Figures and Tables

**Table 1:** Demographic characteristics of patients.

	Peat intervention group (*n* = 22) mean ± SD	Control group (*n* = 19) mean ± SD	*p* value^†^
Age (years)	66.41 ± 5.93	65.16 ± 9.31	0.618
Body mass index	23.85 ± 2.42	25.07 ± 3.22	0.187
Kellgren-Lawrence grade ^*∗*^ (grade 1/2/3)	10/7/5	6/7/6	0.482
Gender (M/F)	8/14	2/17	0.075

^*∗*^All patients participating in the study had the same Kellgren-Lawrence osteoarthritis grade on both knees. ^†^Age and body mass index comparison between two groups was done by the Mann-Whitney test. Kellgren-Lawrence grade and gender comparison between two groups were done by the chi-squared test.

**Table 2:** Changes of VAS score and serum COMP in peat intervention and control group.

	Group	Before intervention		After intervention		*p* value (intragroup analysis)^‡^
		Mean ± SD	*p* value (intergroup analysis)^†^	Mean ± SD	*p* value (intergroup analysis)^†^	
VAS score	Peat group (*n* = 22)	6.000 ± 1.633	0.605	3.409 ± 1.333	0.066	<0.001^*∗*^
	Control group (*n* = 19)	5.737 ± 1.593		4.421 ± 1.953		<0.001^*∗*^

VAS score difference	Peat group (*n* = 22)			2.591 ± 1.333	<0.001^*∗*^	
	Control group (*n* = 19)			1.316 ± 0.885		

Serum COMP (ng/ml)	Peat group (*n* = 22)	2.872 ± 0.937	0.612	2.706 ± 0.627	0.426	0.310
	Control group (*n* = 19)	3.038 ± 1.120		2.921 ± 1.005		0.156

SD: standard deviation. ^*∗*^*p* < 0.05, significant difference at intergroup and intragroup comparing. ^†^Comparison between peat intervention and control groups, Mann–Whitney test. ^‡^Comparison between before- and afterintervention in each group, Wilcoxon signed-rank test.

**Table 3:** Changes of velocity, gait cycle time, and cadence in peat intervention and control groups.

	Group	Before intervention		After intervention		*p* value (intragroup analysis)^‡^
		Mean ± SD	*p* value (intergroup analysis)^†^	Mean ± SD	*p* value (intergroup analysis)^†^	
Velocity (m/s)	Peat group (*n* = 22)	0.781 ± 0.144	0.876	0.873 ± 0.106	0.006^*∗*^	0.002^*∗*^
	Control group (*n* = 19)	0.772 ± 0.195		0.727 ± 0.191		0.180

Gait cycle time (second)	Peat group (*n* = 22)	1.344 ± 0.240	0.977	1.297 ± 0.248	0.634	0.058
	Control group (*n* = 19)	1.346 ± 0.168		1.330 ± 0.188		0.542

Cadence (steps/min)	Peat group (*n* = 22)	91.774 ± 13.526	0.694	95.284 ± 14.652	0.368	0.035^*∗*^
	Control group (*n* = 19)	90.327 ± 9.756		91.675 ± 10.610		0.440

SD: standard deviation. ^*∗*^*p* < 0.05, significant difference at intergroup and intragroup comparison. ^†^Comparison between peat intervention and control groups, Mann–Whitney test. ^‡^Comparison between before- and afterintervention in each group, Wilcoxon signed-rank test.

**Table 4:** Range of motion of knee joint at frontal plane in peat intervention and control groups.

	Group	Before intervention		After intervention		*p* value (intragroup analysis)^‡^
		Mean ± SD	*p* value (intergroup analysis)^†^	Mean ± SD	*p* value (intergroup analysis)^†^	
Knee varus/valgus range of motion (degree)	Peat group (*n* = 44)^*∗∗*^	11.455 ± 6.007	0.298	8.439 ± 3.605	0.082	0.006^*∗*^
	Control group (*n* = 38)^*∗∗*^	10.180 ± 4.838		10.144 ± 4.926		0.975

SD: standard deviation. ^*∗*^*p* < 0.05, significant difference at intergroup and intragroup comparing. ^*∗∗*^Because the data are about both knees, the number of participants doubled. ^†^Comparison between peat intervention and control group, Student's *t* test. ^‡^Comparison between before- and afterintervention, paired *t* test.

## Data Availability

The data used to support the findings of this study are available from the corresponding author upon request.

## References

[1] Loeser R. F., Goldring S. R., Scanzello C. R., Goldring M. B. (2012). Osteoarthritis: a disease of the joint as an organ. *Arthritis & Rheumatism*.

[2] Bijlsma J. W., Berenbaum F., Lafeber F. P. (2011). Osteoarthritis: an update with relevance for clinical practice. *The Lancet*.

[3] Kim Y.-J., Kim S.-H., Lee H. J. (2018). Infectious adverse events following acupuncture: clinical progress and microbiological etiology. *Journal of Korean Medical Science*.

[4] Hurley M. V., Scott D. L., Rees J., Newham D. J. (1997). Sensorimotor changes and functional performance in patients with knee osteoarthritis. *Annals of the Rheumatic Diseases*.

[5] Salaffi F., Carotti M., Stancati A., Grassi W. (2005). Health-related quality of life in older adults with symptomatic hip and knee osteoarthritis: a comparison with matched healthy controls. *Aging Clinical and Experimental Research*.

[6] Wang X., Hunter D. J., Jin X., Ding C. (2018). The importance of synovial inflammation in osteoarthritis: current evidence from imaging assessments and clinical trials. *Osteoarthritis and Cartilage*.

[7] Loeser R. F. (2013). Aging processes and the development of osteoarthritis. *Current Opinion in Rheumatology*.

[8] Loeser R. F. (2013). Osteoarthritis year in review 2013: biology. *Osteoarthritis and Cartilage*.

[9] Silva H. G. P. V. D., Cliquet Junior A., Zorzi A. R., Miranda J. B. D. (2012). Modificações biomecânicas na marcha de indivíduos com osteoartrite medial do joelho. *Acta Ortopédica Brasileira*.

[10] Hurwitz D. E., Ryals A. B., Case J. P., Block J. A., Andriacchi T. P. (2002). The knee adduction moment during gait in subjects with knee osteoarthritis is more closely correlated with static alignment than radiographic disease severity, toe out angle and pain. *Journal of Orthopaedic Research*.

[11] Christensen R., Bartels E. M., Astrup A., Bliddal H. (2007). Effect of weight reduction in obese patients diagnosed with knee osteoarthritis: a systematic review and meta-analysis. *Annals of the Rheumatic Diseases*.

[12] Messier S. P., Mihalko S. L., Legault C. (2013). Effects of intensive diet and exercise on knee joint loads, inflammation, and clinical outcomes among overweight and obese adults with knee osteoarthritis. *JAMA*.

[13] Zhang W., Nuki G., Moskowitz R. W. (2010). OARSI recommendations for the management of hip and knee osteoarthritis. *Osteoarthritis and Cartilage*.

[14] Bannuru R. R., Schmid C. H., Kent D. M., Vaysbrot E. E., Wong J. B., McAlindon T. E. (2015). Comparative effectiveness of pharmacologic interventions for knee osteoarthritis. *Annals of Internal Medicine*.

[15] Derry S., Moore R. A., Rabbie R. (2012). Topical NSAIDs for chronic musculoskeletal pain in adults. *Cochrane Database of Systematic Reviews*.

[16] McAlindon T. E., Bannuru R. R., Sullivan M. C. (2014). OARSI guidelines for the non-surgical management of knee osteoarthritis. *Osteoarthritis and Cartilage*.

[17] Weinstein A. M., Rome B. N., Reichmann W. M. (2013). Estimating the burden of total knee replacement in the United States. *The Journal of Bone and Joint Surgery-American Volume*.

[18] Gutenbrunner C., Bender T., Cantista P., Karagülle Z. (2010). A proposal for a worldwide definition of health resort medicine, balneology, medical hydrology and climatology. *International Journal of Biometeorology*.

[19] Fraioli A., Mennuni G., Fontana M (2018). Efficacy of spa therapy, mud-pack therapy, balneotherapy, and mud-bath therapy in the management of knee osteoarthritis. A systematic review. *BioMed Research International*.

[20] Gorham E. (1957). The development of peat lands. *The Quarterly Review of Biology*.

[21] Beer A. M., Junginger H. E., Lukanov J., Sagorchev P. (2003). Evaluation of the permeation of peat substances through human skin in vitro. *International Journal of Pharmaceutics*.

[22] van Rensburg C. E. J. (2015). The antiinflammatory properties of humic substances: a mini review. *Phytotherapy Research*.

[23] Altman R., Asch E., Bloch D. (1986). Development of criteria for the classification and reporting of osteoarthritis: classification of osteoarthritis of the knee. *Arthritis & Rheumatism*.

[24] Kellgren J. H., Lawrence J. S. (1957). Radiological assessment of osteo-arthrosis. *Annals of the Rheumatic Diseases*.

[25] Jeonnam Institute of Natural Resoures Research (2018). The base study to discover and to commercialize for the resources of sea healing to activate marine industry.

[26] Price D. D., McGrath P. A., Rafii A., Buckingham B. (1983). The validation of visual analogue scales as ratio scale measures for chronic and experimental pain. *Pain*.

[27] Verma P., Dalal K. (2013). Serum cartilage oligomeric matrix protein (COMP) in knee osteoarthritis: a novel diagnostic and prognostic biomarker. *Journal of Orthopaedic Research*.

[28] Watt F. E. (2018). Osteoarthritis biomarkers: year in review. *Osteoarthritis and Cartilage*.

[29] Ortega E., Gálvez I., Hinchado M. D., Guerrero J., Martín-Cordero L., Torres-Piles S. (2017). Anti-inflammatory effect as a mechanism of effectiveness underlying the clinical benefits of pelotherapy in osteoarthritis patients: regulation of the altered inflammatory and stress feedback response. *International Journal of Biometeorology*.

[30] Sarsan A., Akkaya N., Özgen M., Yildiz N., Atalay N. S., Ardic F. (2012). Comparing the efficacy of mature mud pack and hot pack treatments for knee osteoarthritis. *Journal of Back and Musculoskeletal Rehabilitation*.

[31] Güngen G., Ardic F., Fιndıkoğlu G., Rota S. (2012). The effect of mud pack therapy on serum YKL-40 and hsCRP levels in patients with knee osteoarthritis. *Rheumatology International*.

[32] Odabasi E., Turan M., Erdem H., Tekbas F. (2008). Does mud pack treatment have any chemical effect? A randomized controlled clinical study. *The Journal of Alternative and Complementary Medicine*.

[33] Tenti S., Fioravanti A., Guidelli G. M., Pascarelli N. A., Cheleschi S. (2014). New evidence on mechanisms of action of spa therapy in rheumatic diseases. *Tang [Humanitas Medicine]*.

[34] Bender T., Nagy G., Barna I., Tefner I., Kádas É., Géher P. (2007). The effect of physical therapy on beta-endorphin levels. *European Journal of Applied Physiology*.

[35] Schepetkin I. A., Khlebnikov A. I., Ah S. Y. (2003). Characterization and biological activities of humic substances from mumie. *Journal of Agricultural and Food Chemistry*.

[36] Odabasi E., Gul H., Macit E., Turan M., Yildiz O. (2007). Lipophilic components of different therapeutic mud species. *The Journal of Alternative and Complementary Medicine*.

[37] Hunter D. J., Nevitt M., Losina E., Kraus V. (2014). Biomarkers for osteoarthritis: current position and steps towards further validation. *Best Practice & Research Clinical Rheumatology*.

[38] Kiliçoğlu O., Dönmez A., Karagülle Z., Erdoğan N., Akalan E., Temelli Y. (2010). Effect of balneotherapy on temporospatial gait characteristics of patients with osteoarthritis of the knee. *Rheumatology International*.

[39] van der Esch M., Steultjens M., Harlaar J. (2008). Varus–valgus motion and functional ability in patients with knee osteoarthritis. *Annals of the Rheumatic Diseases*.

[40] van der Esch M., Steultjens M., Harlaar J., Wolterbeek N., Knol D. L., Dekker J. (2008). Knee varus-valgus motion during gait—a measure of joint stability in patients with osteoarthritis?. *Osteoarthritis and Cartilage*.

[41] Mündermann A., Dyrby C. O., Hurwitz D. E., Sharma L., Andriacchi T. P. (2004). Potential strategies to reduce medial compartment loading in patients with knee osteoarthritis of varying severity: reduced walking speed. *Arthritis & Rheumatism*.

[42] Robbins S. M. K., Maly M. R. (2009). The effect of gait speed on the knee adduction moment depends on waveform summary measures. *Gait & Posture*.

[43] Galzigna L., Moretto C., Lalli A. (1996). Physical and biochemical changes of thermal mud after maturation. *Biomedicine & Pharmacotherapy*.

[44] Tuncer T., Cay H. F., Kacar C. (2012). Evidence-based recommendations for the management of knee osteoarthritis: a consensus report of the Turkish league against rheumatism. *Turkish Journal of Rheumatology*.

